# Posterior Cervical Glomus Tumor Mimicking Neurogenic Occipital Pain

**DOI:** 10.7759/cureus.51514

**Published:** 2024-01-02

**Authors:** Diogo Luz, Carlos Casimiro, Cátia Gradil

**Affiliations:** 1 Neurosurgery, Hospital Garcia de Orta, Almada, PRT

**Keywords:** pain management, neurosurgery, rare tumors, neurogenic pain, glomus tumor

## Abstract

Glomus tumors, typically localized in digits, palms, and soles, rarely occur in the posterior cervical region. This case report describes a unique presentation of an epithelioid glomus tumor in a 49-year-old male with a history of progressive occipital headaches. A 49-year-old male, referred with a five-year history of worsening occipital headaches, presented a palpable lesion in the right suboccipital area. MRI identified a 2.3 cm subcutaneous lesion adjacent to the right occipital artery, raising initial suspicion of a schwannoma. Subsequent excisional biopsy unveiled an unexpected diagnosis - an epithelioid glomus tumor. The rarity of glomus tumors in the posterior cervical region, coupled with their potential to mimic neurogenic tumors like schwannomas, underscores the diagnostic complexity. This encounter of a glomus tumor in an uncommon posterior cervical location serves as a pertinent reminder for neurosurgeons to consider atypical differentials. This case underscores the need for heightened clinical vigilance when faced with unusual presentations in neurosurgical practice.

## Introduction

Glomus tumors, originating from the glomus body, constitute a distinctive clinical entity with a proclivity for the digits, palms, and soles of the feet. These tumors manifest as localized pain and tenderness, contributing to their misdiagnosis due to their clinical overlap with other conditions [1-3].

This article presents a unique case of a patient with posterior cervical pain, suspected to be of neurogenic origin and initially thought to be associated with a schwannoma or neurofibroma. The case underscores the difficulties surrounding the accurate diagnosis of extradigital glomus tumors and highlights the importance of considering glomus tumors in the differential diagnosis of neurogenic pain.

## Case presentation

**Figure 1:**
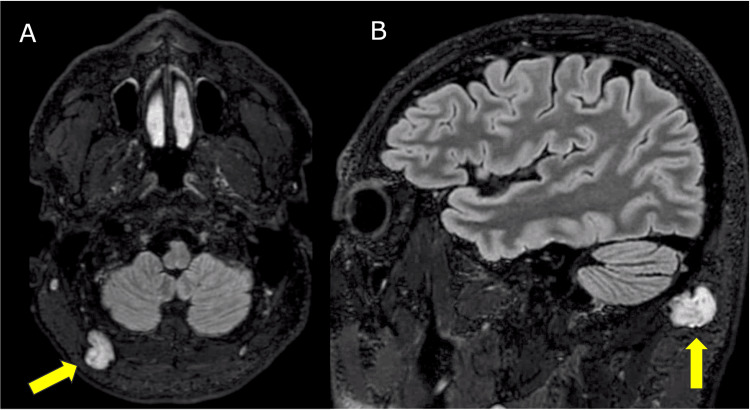
T2 FLAIR axial (A) and sagittal (B) MRI images highlighting a posterior subcutaneous hyperintense lesion. The figure illustrates the T2 FLAIR axial (A) and sagittal (B) MRI images highlighting a posterior subcutaneous hyperintense lesion that borders the right occipital artery, as indicated by the yellow arrows. FLAIR: fluid-attenuated inversion recovery.

**Figure 2:**
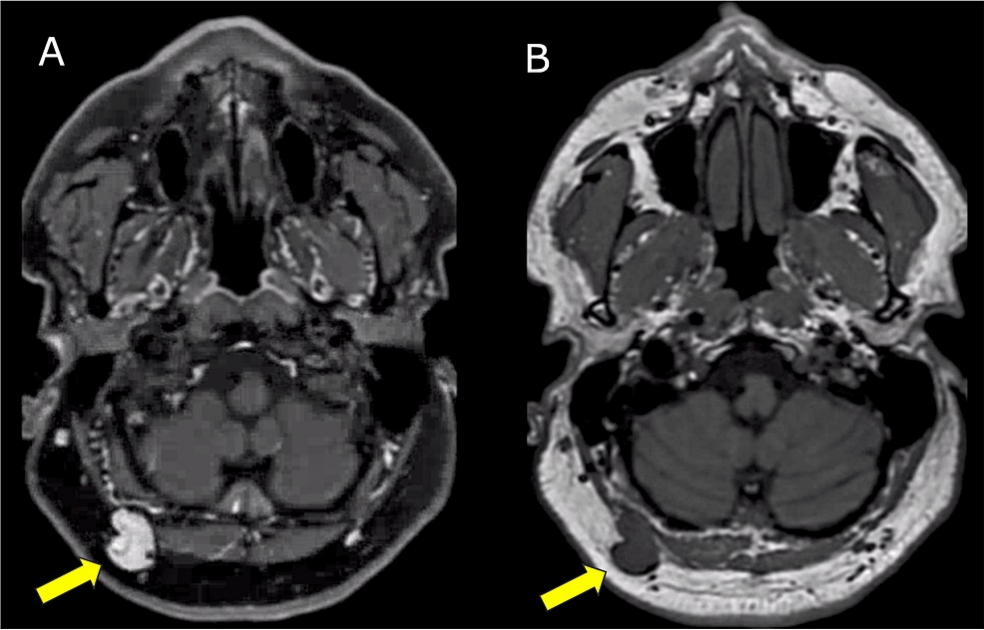
T1-weighted MRI images before (B) and after (A) gadolinium administration showing homogenous enhancement of the lesion. The figure illustrates T1-weighted MRI images before (B) and after (A) gadolinium administration showing a homogenous gadolinium enhancement of the lesion, as highlighted by the yellow arrows.

A 49-year-old male presented with a five-year history of progressively aggravated occipital headaches, reaching a peak of eight on the visual analog scale (VAS), localized to the right occipital region, and exacerbated by pressure. Clinical examination revealed a palpable lesion in the right suboccipital area, with pain radiating to the adjacent scalp regions. Magnetic resonance imaging (MRI) investigation unveiled a 2.3 cm subcutaneous lesion adjacent to the right occipital artery and greater occipital nerve (Figures 1, 2). Radiologically, the lesion raised suspicion of a schwannoma or neurofibroma related to the greater occipital nerve. Consequently, an excisional biopsy was performed, successfully removing a well-circumscribed, soft lesion closely associated with the right occipital artery.

Histopathological examination of the excised tissue revealed a well-demarcated, expansive neoplasm composed of three distinct cellular components. The first component consisted of round cells with ill-defined boundaries, characterized by round, hyperchromatic, and homogenous nuclei with inconspicuous nucleoli and eosinophilic cytoplasm. The second component was composed of spindle-shaped cells resembling smooth muscle cells. The third component included vascular spaces, indicating a significant vascular component within the tumor. Notably, the lesion was negative for both typical and atypical mitoses, as well as for necrosis, suggesting a lower likelihood of aggressive behavior. The immunohistochemical profile further supported these findings. The lesion tested positive for vimentin, actin (smooth muscle-specific 1A4 and HHF-35), caldesmon, calponin, CD34, and type IV collagen, while being negative for CK AE1/AE3, CD31, S100, CD117, chromogranin A, synaptophysin, CD20, CD45, desmin, WT1, and HMB45. These immunohistochemical characteristics, in conjunction with the histomorphological features, aligned with those typically observed in glomus tumors, leading to the conclusive diagnosis of a glomus tumor.

The patient underwent a one-year follow-up, during which no evidence of tumor recurrence or complications was observed.

## Discussion

This case of a 49-year-old male patient, marked by a prolonged history of occipital headaches and a palpable lesion in the right occipital region, casts a spotlight on the diverse array of manifestations and diagnostic intricacies inherent to glomus tumors. What sets this case apart is the rarity of both its location and the initial differential diagnosis.

This particular type of localized pain has often led to misdiagnoses resembling neuropathic pain syndromes, propelling glomus tumors into consideration as potential underlying etiologies [2]. The patient's enduring experience of occipital headaches over a five-year span, along with the gradual worsening of symptoms, underscores the insidious nature of glomus tumors. These initial symptoms may be subtle and nonspecific, making diagnosis challenging. The key attribute of the lesion being palpable, accompanied by localized pain radiating to neighboring areas, resonates with the defining characteristics of glomus tumors [3].

MRI emerged as an important diagnostic tool in characterizing the subcutaneous lesion situated in the right suboccipital region. This lesion, positioned adjacent to the right occipital artery and greater occipital nerve, prompted an initial differential diagnosis that encompassed schwannoma or neurofibroma. The rationale for proceeding with an excisional biopsy was well-founded, given the dual objectives of confirming the diagnosis and mitigating the patient's debilitating symptoms.

Contemporary research increasingly acknowledges the intricate diagnostic landscape of glomus tumors, a result of their relatively infrequent incidence and diverse anatomical occurrences [2,4]. Glomus tumors tend to favor acral regions, such as fingertips, where the abundantly found modified smooth muscle cells constitute glomus bodies [2]. However, the unique aspect here is the presence of this tumor in the posterior cervical region, a location scarcely reported in existing literature [3,5]. In its comprehensive analysis of 272 reported cases, Beaton and Davis identified only a single instance of a scalp glomus tumor, which regrettably lacked detailed description and clinical context [4]. Delving into more recent literature in search of scalp glomus tumors reveals the scarcity of such cases. A letter by Yanagi and Matsumura briefly mentions one case, yet provides limited information regarding the patient's clinical presentation and outcome [5]. Another notable instance dates back to 1997, documented in a report covering three cases of extradigital glomus tumors. Among these cases, one patient presented with a major occipital nerve glomus tumor, underscoring the rarity of this occurrence [3].

The unique convergence of this case with prior research highlights the potential of glomus tumors to incite neuralgia-like symptoms. Muller documented instances of neuralgia within territories innervated by the greater occipital nerve, obturator nerve, and anterior brachial cutaneous nerve, revealing the capacity of glomus tumors to mimic neuropathic pain scenarios [3]. Similarly, isolated cases linking sciatica and radial nerve neuralgia to glomus tumors have also been reported, and emphasize their ability to simulate neuropathic pain scenarios [6].

The successful resolution of this patient's case through surgical excision spotlights the criticality of total tumor removal for achieving enduring relief [3]. Regarding recurrence, this case also aligns with prior investigations suggesting a relatively low recurrence incidence following surgical intervention [3].

## Conclusions

The case we report represents a rare and intriguing clinical entity. The uniqueness lies in the tumor's atypical location and its presentation, which initially mimicked neurogenic pain, leading to a presumptive misdiagnosis. Furthermore, the distinct histopathological and immunohistochemical findings of this tumor contribute to the limited but growing body of literature on the various presentations of glomus tumors.

Ultimately, this case serves as a reminder of the diversity of pathologies that can present in routine clinical practice and the necessity of maintaining a broad diagnostic perspective when evaluating atypical clinical presentations.
